# Evaluating the efficacy and safety of intravesical chemotherapies for non-muscle invasive bladder cancer: a network meta-analysis

**DOI:** 10.18632/oncotarget.12856

**Published:** 2016-10-24

**Authors:** Chuanjun Zhuo, Xubin Li, Hongqing Zhuang, Shunli Tian, Hailong Cui, Ronghuan Jiang, Chuanxin Liu, Ran Tao, Xiaodong Lin

**Affiliations:** ^1^ Department of Psychological Medicine, Wenzhou Seventh People's Hospital, Wenzhou, Zhejiang, 325005, China; ^2^ Department of Psychological Medicine, Jining Medical University, Jining, Shandong, 272067, China; ^3^ Department of Psychological Medicine, Tianjin Anding Hospital, Tianjin, 300222, China; ^4^ Department of Radiotherapy, Tianjin Cancer Institute and Hospital, Tianjin, 30000, China; ^5^ Department of Geriatrics, Tianjin Medical University General Hospital, Tianjin, 300075, China; ^6^ Intensive Care Unit, Tianjin Medical University General Hospital, Tianjin, 300075, China; ^7^ Department of Psychological Medicine, Chinese PLA (People's Liberation Army) General Hospital; Chinese PLA (People's Liberation Army) Medical School, Beijing, 100853, China; ^8^ Beijing Shijian Integrated Medicine Science Institute, Beijing, 100700, China

**Keywords:** non-muscle invasive bladder cancer, intravesical therapy, efficacy and safety, tumor recurrence, tumor progression

## Abstract

Various intravesical therapies have been introduced into clinical practices for controlling non-muscle invasive bladder cancer (NMIBC). However, evidence with respect to the efficacy and safety of those intravesical therapies is very limited. Hence, we present a network meta-analysis in order to address this limitation in the current literature. The primary outcomes were the risk of tumor recurrence (TR), tumor progression (TP) and disease-specific mortality (DM). Secondary outcomes included the risk of fever, cystitis and haematuria. Conventional pair-wise and network meta-analysis were both performed for each endpoint. The surface under the cumulative ranking curve (SUCRA) was incorporated in our analysis for ranking the corresponding intravesical instillation interventions. In total, 23 randomized clinical trials (RCTs) were finally included in our study after irrelevant papers were screened out. Results of network meta-analysis suggested that Epirubicin (EPI) was less preferable than Bacille Calmette Guerin (BCG), BCG+EPI, BCG+ Isoniazid (INH), BCG+ Mytomicin C (MMC), Gemcitabine (GEM) and MMC with respect to TR. As suggested by the corresponding ranking probabilities and SUCRA, incorporating EPI or MMC into BCG may enhance the efficacy of BCG monotherapy.

## INTRODUCTION

A large number of populations suffer from bladder cancer which resulted in 380,000 cases and 150,000 deaths over the world in 2008 [1]. Bladder cancer is classified as either muscle-invasive bladder cancer (MIBC) or non-muscle-invasive bladder cancer (NMIBC) according to disease severity (high or low) and the extent of permeation [2]. Approximately 70% of bladder cancers are NMIBC which can be further classified into carcinoma-in-situ (CIS), papillary tumor (Ta) and those that are able to invade the lamina propria rather than the detrusor muscle (T1) [3]. Transurethral resection (TUR) is a standard treatment for NMIBC [4]. However, patients treated with TUR are often accompanied with a high tumor recurrence (TR) rate ranging from 50% to 70% as well as a high tumor progression (TP) rate between 10% and 20% over a period of 2–5 years [5, 6]. Hence, a few intravesical therapies which instill chemotherapeutic agents into bladder have been developed to supplement TUR.

Bacille Calmette-Guerin (BCG) is one of the most popular chemotherapeutic agents that have been incorporated into intravesical therapies. Adjuvant treatments such as intravesical instillations of BCG are appropriate for those NMIBC patients with a high risk of recurrence [4, 7]. Unfortunately, about one third of NMIBC patients eventually experience side effects resulted from BCG, including mild dysuria [8]. Recently, mytomicin C (MMC) is introduced into intravesical therapies in order to overcome the corresponding side effects [9]. MMC is effective for non-severe bladder cancer and it is usually administered by using multiple infusions which is able to produce response rates ranging between 40% and 50% [6].

Apart from the above mentioned chemotherapeutic agents, epirubicin (EPI) is another one which is applied in conjunction with intravesical therapies and the popularity of EPI has been increased in the Europe and Japan [10]. EPI belongs to the anthracyclines family and it was developed to reduce adverse effects resulted from chemotherapeutic agents without significant compensation of antitumor effects [11]. Antitumor effects are triggered by EPI which is able to interfere with DNA synthesis and EPI is associated with a relatively low risk of side effects particularly in patients with Ta or T1 bladder cancer [12].

On the other hand, new agents such as gemcitabine (GEM) are well adapted for intravesical instillation treatment and they are usually incorporated into systemic therapies for advanced invasive bladder cancer [13]. GEM is an antimetabolite pyrimidine base analogue and it has been widely used as a chemotherapeutic agent for pancreatic and lung cancer [14, 15]. Various experimental studies *in vitro* and *in vivo* have been conducted to verify the effects of GEM on bladder cancer and its efficacy has been validated in an *in vitro* model [16].

As suggested by published data, intravesical chemotherapies (both monotherapy and polytherapy), namely BCG, EPI, MMC and GEM, are effective for preventing TR and TP [9, 17–19]. However, a comprehensive analysis of both efficacy and safety of these therapies has not been disclosed by the current literature due to the lack of evidence. For this purpose, we designed this network meta-analysis in order to determine the relative efficacy and safety of several intravesical chemotherapies: BCG, BCG+EPI, BCG+ Isoniazid (INH), BCG+MMC, EPI, GEM and MMC.

## RESULTS

### Baseline characteristics of the included studies

**Figure 1:**
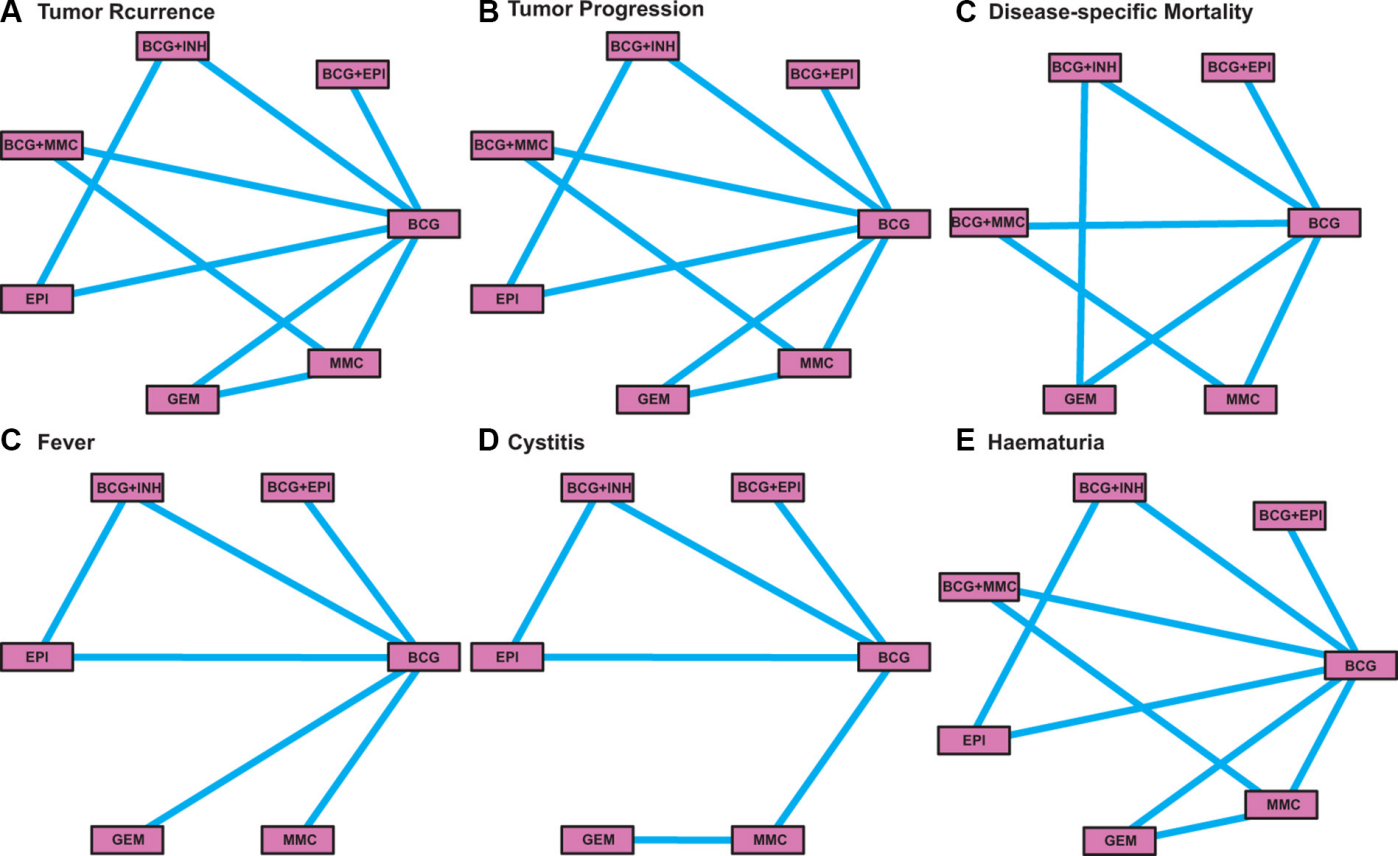
The network plot of intravesical chemotherapies included in this meta-analysis

In total 23 randomized clinical trials (RCTs) were finally included in our study after screening out irrelevant papers (Table 1) [6, 20–41]. Among the total 5,822 subjects with follow-up periods ranging from 1 to 7.2 years, 2,399 (41.21%) individuals were treated with BCG and 1,064 (18.28%) individuals were treated with MCC (Figure 1). There are 2 three-arm trials and 21 two-arm trials which contain a total of seven comparisons. Moreover, there are 22 studies in which TR was assessed as the primary endpoint. TP and disease-specific mortality (DM) was assessed by 20 and 12 trials, respectively. Secondary endpoints including fever, cystitis and haematuria were researched in 9, 8 and 11 studies, respectively. The quality of included studies was assessed by using the Jadad scale (Supplementary Table S1).

**Table 1:** The main characteristics of included studies

Author	Year	Follow-up (yr)	Size	Age	Male (%)	Intervention	Induction	Outcomes					
								TR	TP	DM	Fever	Cystitis	Haematuria
Solsona	2015	7.1	407	67	89.9	MMC+BCG	30 mg + 81 mg Connaught strain	44	26	10			
						BCG	81 mg Connaught strain	68	24	15			
Gontero	2013	1	120	67.4	85.8	BCG	27 mg Connaught strain	14	3				
						GEM	2000 mg	16	5				
Järvinen	2012	7.2	68	68	80.9	MMC	-	35	14	12			
						MMC+BCG	-	19	8	8			
Oosterlinck	2011	4.7	96	69	86.5	MMC+BCG	40 mg + TICE 5 × 10^8 CFU	23	2	0			
						BCG	TICE 5 × 10^8 CFU	26	5	6			
Hinotsu	2011	2	115	NR	90.4	BCG	81 mg Connaught strain	19	3				69
						EPI	40 mg	22	7				
Sylvester	2010	9.2	837	67	NR	EPI	50 mg	147	24	19			
						BCG	TICE 5 × 10^8 CFU	103	19	9			
						BCG+INH	TICE 5 × 10^8 CFU + 300 mg	110	23	10			
Porena	2010	3.7	64	69.4	84.4	BCG	TICE 5 × 10^8 CFU	9	5		2		
						GEM	2000 mg	17	10		0		
Di Lorenzo	2010	1.3	80	70.4	61.2	GEM	2000 mg	21	7		1		2
						BCG	81 mg Connaught strain	35	13		3		5
Addeo	2010	3	120	66.4	85.3	MMC	40 mg	22	10			12	4
						GEM	2000 mg	15	6			3	2
Cai	2008	1.3	161	NR	85.7	EPI+BCG	80 mg + TICE 5 × 10^8 CFU	34	2				
						BCG	TICE 5 × 10^8 CFU	40	4				
Ojea	2007	4.4	430	64.5	87	BCG	27 mg Pasteur	88	32	8			
						MMC	30 mg	58	141	7			
Friedrich	2007	2.9	495	67.4	80.2	BCG	RIVM 2 × 10^8 CFU	41			15		19
						MMC	20 mg	62			8		15
de Reijke	2005	5.6	168	NR	92	BCG	81 mg Connaught strain	9		9	6	15	33
						EPI	50 mg	21		13	0	7	23
Cheng	2005	5.1	209	69.9	71.2	BCG	81 mg Connaught strain	30	9	13			
						EPI	50 mg	59	16	7			
Kaasinen	2003	4.7	304	70.5	80.3	MMC+BCG	40 mg			13			
						BCG	120 mg Connaught			10			
Di Stasi	2003	3.6	108	NR	73	BCG	81 mg Pasteur	19	6		7	24	26
						MMC	40 mg	46	14		0	22	14
van der Meijden	2001	3.5	957	66	77	EPI	50 mg	142	19	12	34	82	45
						BCG	TICE 5 × 10^8 CFU	98	9	5	38	111	93
						BCG+INH	TICE 5 × 10^8 CFU + 300 mg	100	15	8	72	113	78
Bilen	2000	1.5	41	55	95.1	BCG	81 mg Connaught strain	4	2	0	3		8
						EPI+BCG	50 mg and 81 mg Connaught strain	3	1	0	2		4
Ali-El-Dein	1999	2.5	139	58.2	77.4	EPI+BCG	50 mg and 150 mg Pasteur strain	7	3		0	18	0
						BCG	150 mg Pasteur strain	12	5		3	36	4
Witjes	1998	7.2	344	NR	81.7	BCG	RIVM strain	76	21	12		30	
						MMC	30 mg	72	12	8		37	
Rintala	1996	2.8	188	68	75.5	MMC	20–40 mg	58	3				
						MMC+BCG	20–40 mg	57	3				
Melekos	1996	2.9	94	NR	NR	BCG	150 mg Pasteur strain	16	5			33	11
						EPI	50 mg	22	7			12	6
Lamm	1995	2.5	377	67	83	BCG	TICE 50 mg	77	15	8	38	19	85
						MMC	20 mg	101	25	12	8	19	61

*yr: year; BCG: Bacille Calmette Guerin; MMC: Mytomicin C; GEM: Gemcitabine; EPI: Epirubicin; INH: Isoniazid; TR: tumor recurrence; TP: tumor progression; DM: disease-specific mortality.

### Pair-wise meta-analysis

The summary odds ratios (ORs) of the corresponding outcomes (TR, TP, DM, fever, cystitis, haematuria) for each direct comparison were calculated. The results of pair-wise meta-analysis were showed in Table 2. Patients with EPI exhibited an increased risk of TR compared to those with BCG (OR = 1.58, 95% CI = 1.23–1.90, *P* < 0.001). By contrast, patients with BCG+MMC exhibited a significantly lower risk of TR than those with BCG (OR = 0.25, 95% CI = 0.10–0.61, *P* = 0.002). Besides that, patients with EPI or MMC exhibited a higher risk of TP than those with BCG (EPI: OR = 1.71, 95% CI = 1.16–2.53, *P* = 0.007; MMC: OR = 3.05, 95% CI = 2.24–4.17, *P* < 0.001). Moreover, patients with MMC were associated with a reduced risk of fever compared to those with BCG (OR = 0.22, 95% CI = 0.13–0.38, *P* < 0.001), while those with BCG + INH were associated with an increased risk of fever in relation to patients with BCG (OR = 1.92, 95% CI = 1.25–2.94, *P* = 0.003). Furthermore, both EPI (OR = 0.64, 95% CI = 0.48–0.86, *P* = 0.003) and BCG + EPI (OR = 0.44, 95% CI = 0.23–0.86, *P* = 0.016) appeared to be more effective than BCG in reducing the risk of cystitis. Finally, patients with MMC (OR = 0.55, 95% CI = 0.41–0.75, *P* = 0.011) and EPI (OR = 0.54, 95% CI = 0.39–0.74, *P* < 0.001) were associated with approximately 45% reduction in the risk of haematuria.

**Table 2:** Pair-wise meta-analyses of direct comparisons between the five drugs

Endpoints	Direct comparisons	*I*^2^	*P_H_* values	OR (95% CI)	*P_OR_* values
**Tumor Recurrence**	MMC vs. BCG	31.58%	0.234	1.07 (0.89, 1.29)	0.593
	GEM vs. BCG	52.37%	0.122	1.01 (0.64, 1.61)	0.829
	EPI vs. BCG	0.00%	0.402	1.58 (1.32, 1.90)	**< 0.001**
	BCG + MMC vs. BCG	0.00%	0.058	0.25 (0.10, 0.61)	0.002
	BCG + EPI vs. BCG	0.00%	0.672	0.76 (0.48, 1.21)	0.254
	BCG + INH vs. BCG	0.00%	0.764	1.07 (0.85, 1.35)	0.547
	MMC vs. GEM	-	-	1.44 (0.68. 3.07)	0.345
	MMC vs. BCG + MMC	0.00%	0.630	1.11 (0.75, 1.64)	0.617
**Tumor Progression**	MMC vs. BCG	91.64%	<.0001	3.05 (2.24, 4.17)	**< 0.001**
	GEM vs. BCG	42.79%	0.182	1.09 (0.55, 2.17)	0.797
	EPI vs. BCG	0.00%	0.351	1.71 (1.16, 2.53)	**0.007**
	BCG + MMC vs. BCG	2.30%	0.312	0.91 (0.52, 1.59)	0.741
	BCG + EPI vs. BCG	0.00%	0.999	0.52 (0.19, 1.44)	0.209
	BCG + INH vs. BCG	0.00%	0.582	1.40 (0.84. 2.32)	0.193
	MMC vs. GEM	-	-	1.64 (0.56, 4.82)	0.371
	MMC vs. BCG + MMC	0.00%	0.852	1.17 (0.50, 2.72)	0.722
**Disease-specific Mortality**	MMC vs. BCG	17.96%	0.303	1.14 (0.66, 1.98)	0.634
	EPI vs. BCG	51.78%	0.102	1.42 (0.89, 2.24)	0.324
	BCG + MMC vs. BCG	22.63%	0.204	0.76 (0.43, 1.34)	0.335
	BCG + EPI vs. BCG	-	-	1.05 (0.06, 17.95)	0.973
	BCG + INH vs. BCG	0.00%	0.646	1.32 (0.65, 2.69)	0.442
	MMC vs. BCG + MMC	-	-	1.05 (0.38, 2.90)	0.925
**Fever**	MMC vs. BCG	0.00%	0.807	0.22 (0.13, 0.38)	**< 0.001**
	EPI vs. BCG	0.00%	0.813	0.40 (0.08, 2.16)	0.289
	BCG + MMC vs. BCG	56.00%	0.132	0.83 (0.51, 1.34)	0.428
	BCG + EPI vs. BCG	0.00%	0.565	0.49 (0.11, 2.11)	0.340
	BCG + INH vs. BCG	-	-	1.92 (1.25, 2.94)	0.003
**Cystitis**	MMC vs. BCG	57.80%	0.096	0.89 (0.62, 1.27)	0.513
	EPI vs. BCG	46.53%	0.166	0.64 (0.48, 0.86)	**0.003**
	BCG + EPI vs. BCG	-	-	0.44 (0.23, 0.86)	**0.016**
	BCG + INH vs. BCG	-	-	1.03 (0.76, 1.41)	0.839
	MMC vs. GEM	-	-	3.93 (1.05, 14.70)	**0.042**
**Haematuria**	MMC vs. BCG	66.89%	0.040	0.55 (0.41, 0.75)	0.011
	GEM vs. BCG	-	-	0.40 (0.07, 2.18)	0.290
	EPI vs. BCG	0.00%	0.627	0.54 (0.39, 0.74)	**0.0001**
	BCG + EPI vs. BCG	0.00%	0.511	0.42 (0.13, 1.31)	0.135
	BCG + INH vs. BCG	-	-	0.85 (0.60, 1.20)	0.357
	MMC vs. GEM	-	-	1.96 (0.35, 11.17)	0.447

*H: heterogeneity; OR: odds ratio; CI: confidence interval; BCG: Bacille Calmette Guerin; MMC: Mytomicin C; GEM: Gemcitabine; EPI: Epirubicin; INH: Isoniazid.

As suggested by Table 2, heterogeneity among studies did not appear to be significant and thereby the fixed-effect model was incorporated in our analysis for some the comparisons. However, significant heterogeneity was presented in some comparisons (GEM vs. BCG, *I²* = 52.37%, in TR; MMC vs. BCG, *I²* = 91.64%, in TP; EPI vs. BCG, *I²* = 51.78%, in DM; BCG+MMC vs. BCG, *I²* = 56.00%, in fever; MMC vs. BCG, *I²* = 57.80%, in cystitis and MMC vs. BCG, *I²* =66.89%, in haematuria) and hence the random-effects model was implemented for these comparisons.

### Network meta-analysis

**Figure 2:**
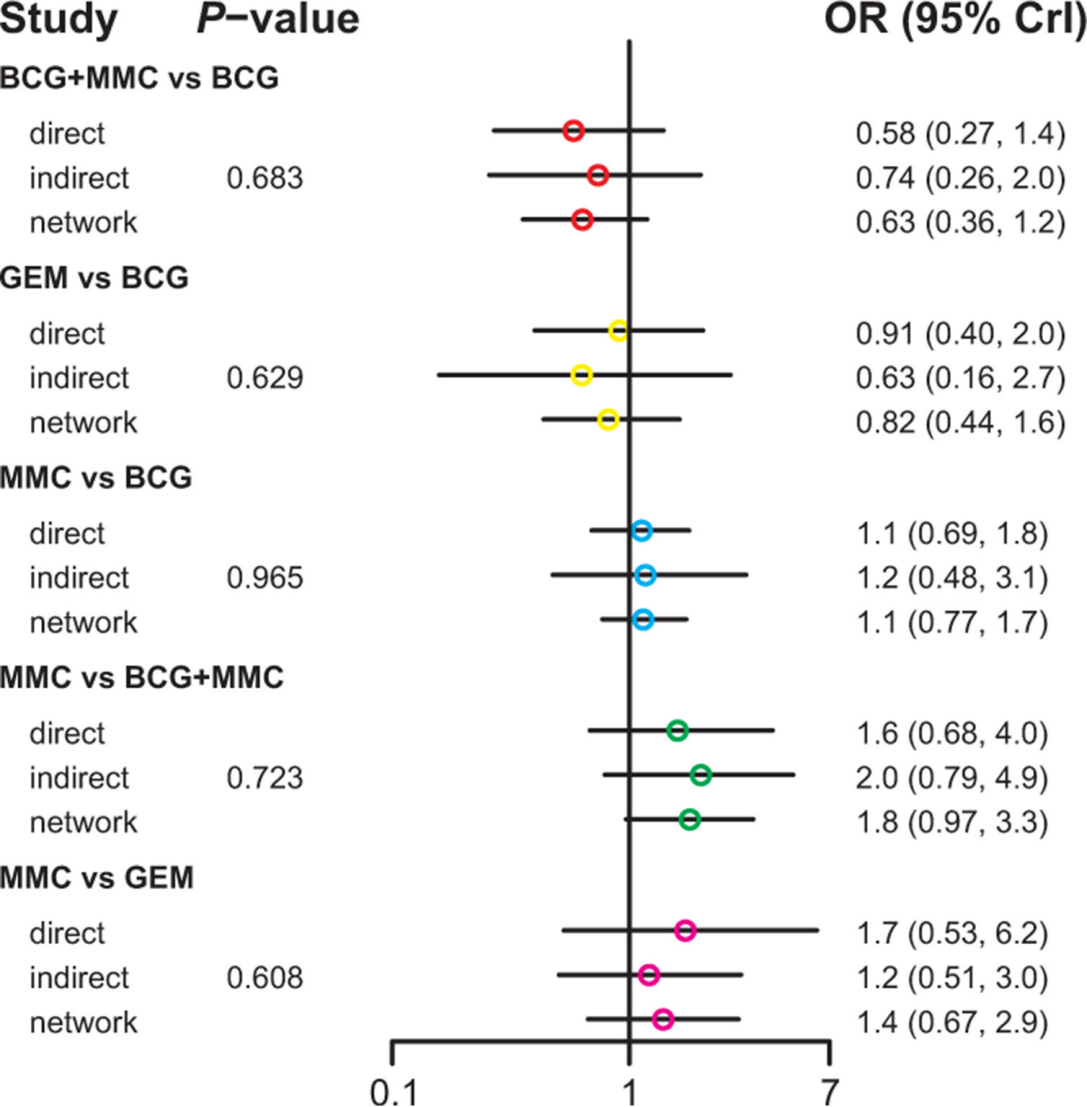
The network node-split plot of tumor recurrence

**Figure 3:**
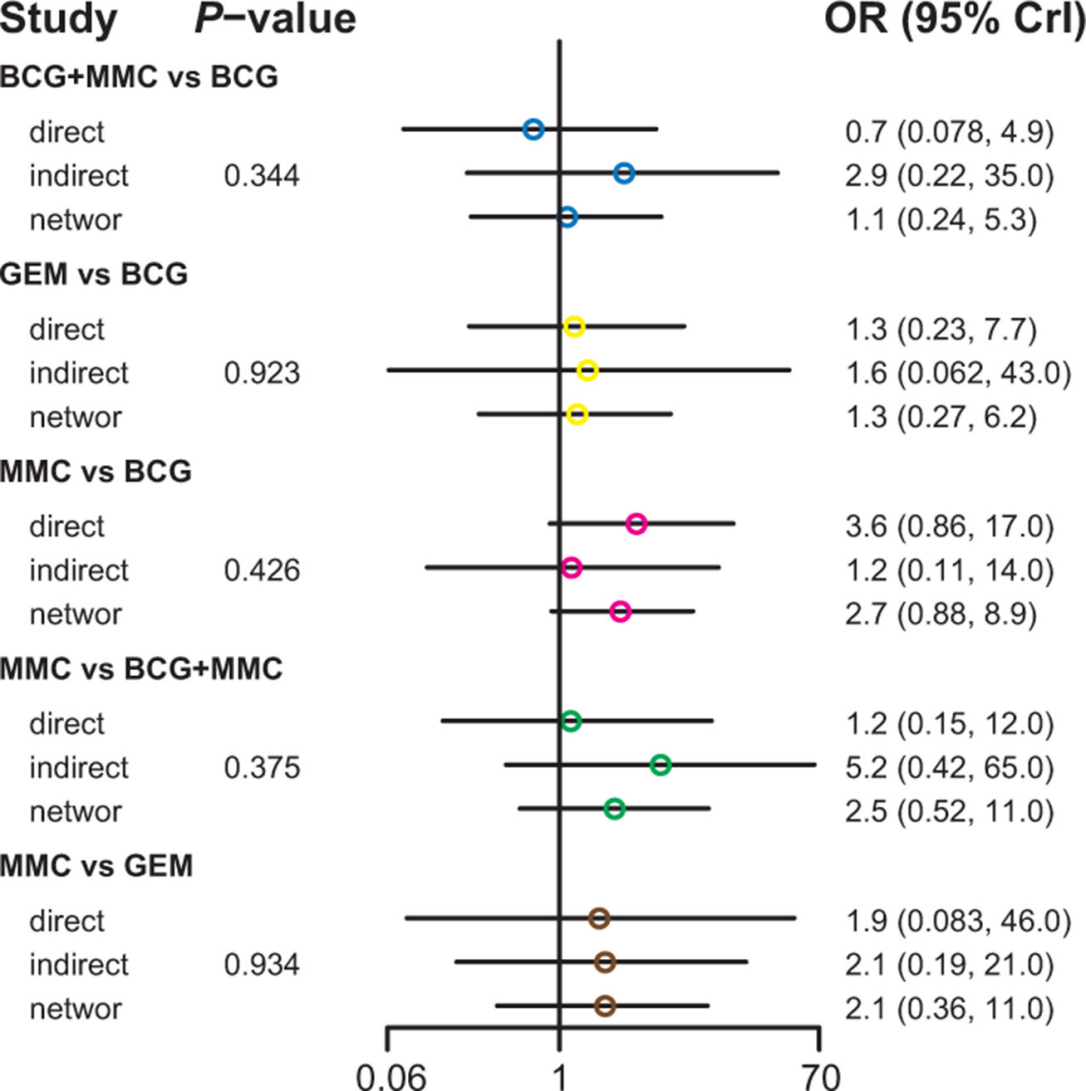
The network node-split plot of tumor progression

**Figure 4:**
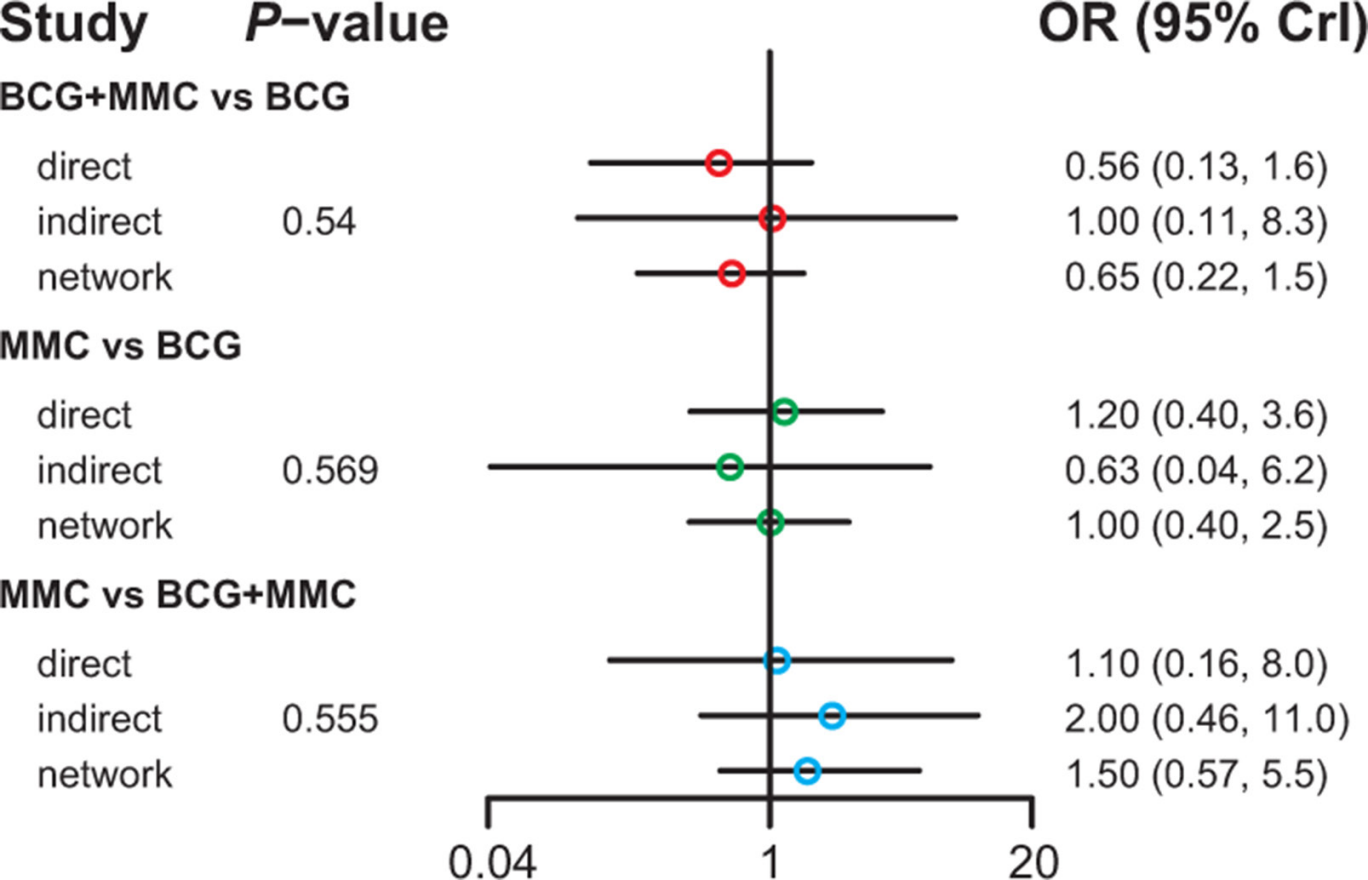
The network node-split plot of disease-specific mortality

**Figure 5:**
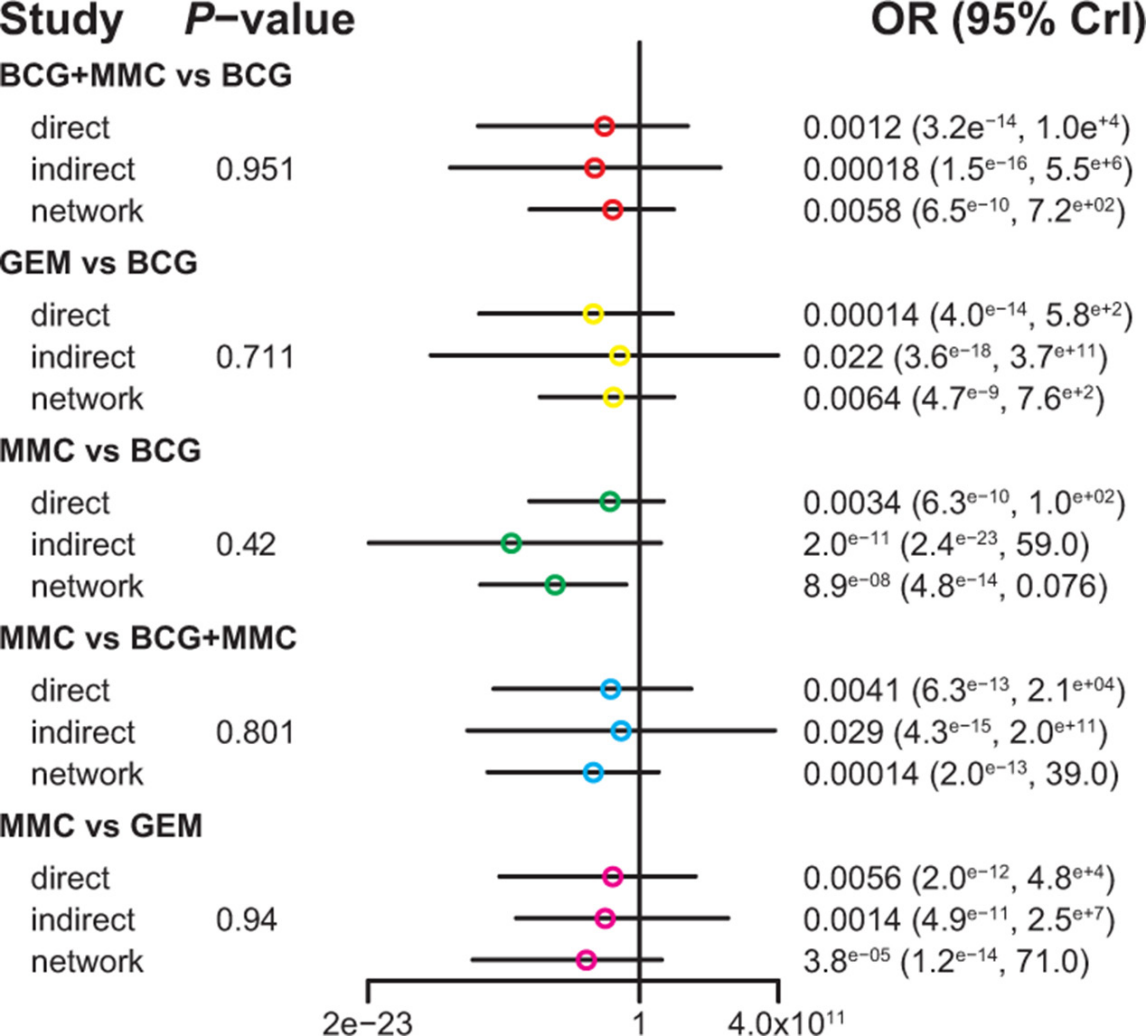
The network node-split plot of haematuria

Table 3 demonstrated the mixed comparisons results by synthesizing all evidence within the network. Patients with all other treatments exhibited a significantly lower risk of TR than those with EPI (OR = 0.40, 95% CrI = 0.25–0.62; OR = 0.25, 95% CrI = 0.10–0.60; OR = 0.51, 95% CrI = 0.26–0.96; OR = 0.26, 95% CrI = 0.11–0.54; OR = 0.33, 95% CrI = 0.15–0.73; OR = 0.45, 95% CrI = 0.25–0.83). On the other hand, patients with MMC exhibited experienced 84% and 91% reduction in the risk of fever compared to those treated with BCG and BCG+INH, respectively (OR = 0.16, 95% CrI = 0.04–0.50; OR = 0.09, 95% CrI = 0.01–0.97). As suggested by Figures 2–5, no significant inconsistency between direct and indirect evidence was presented with respect to TR, TP, DM or haematuria and thereby a consistent model was used in our analysis.

**Table 3:** The efficacy and tolerability of seven treatments according to the network meta-analysis using odds ratios (ORs) and corresponding 95% credible intervals (CrIs)

Tumor Recurrence						
**BCG**	0.63 (0.28, 1.35)	1.27 (0.67, 2.49)	0.64 (0.34, 1.18)	**2.51 (1.62, 4.05)**	0.83 (0.43, 1.63)	1.14 (0.76, 1.73)
1.59 (0.74, 3.57)	**BCG_EPI**	2.03 (0.75, 5.91)	1.02 (0.38, 2.80)	**3.98 (1.66, 10.31)**	1.34 (0.48, 3.78)	1.81 (0.77, 4.59)
0.79 (0.40, 1.49)	0.49 (0.17, 1.34)	**BCG_INH**	0.50 (0.20, 1.20)	**1.96 (1.04, 3.82)**	0.65 (0.25, 1.61)	0.90 (0.41, 1.92)
1.56 (0.84, 2.90)	0.98 (0.36, 2.66)	1.99 (0.83, 5.04)	**BCG_MMC**	**3.90 (1.85, 8.74)**	1.30 (0.54, 3.10)	1.78 (0.96, 3.36)
**0.40 (0.25, 0.62)**	**0.25 (0.10, 0.60)**	**0.51 (0.26, 0.96)**	**0.26 (0.11, 0.54)**	**EPI**	**0.33 (0.15, 0.73)**	**0.45 (0.25, 0.83)**
1.20 (0.61, 2.35)	0.75 (0.26, 2.08)	1.54 (0.62, 4.00)	0.77 (0.32, 1.84)	**3.04 (1.36, 6.86)**	**GEM**	1.37 (0.66, 2.83)
0.87 (0.58, 1.31)	0.55 (0.22, 1.30)	1.12 (0.52, 2.42)	0.56 (0.30, 1.04)	**2.20 (1.20, 4.04)**	0.73 (0.35, 1.51)	**MMC**

Tumor Progression						
**BCG**	0.46 (0.06, 3.07)	1.73 (0.27, 10.98)	1.16 (0.22, 5.92)	2.23 (0.60, 8.60)	1.35 (0.29, 6.27)	2.69 (0.80, 8.99)
2.19 (0.33, 17.18)	**BCG_EPI**	3.75 (0.26, 58.16)	2.57 (0.18, 36.01)	4.89 (0.49, 54.05)	3.01 (0.24, 36.91)	5.92 (0.60, 62.73)
0.58 (0.09, 3.70)	0.27 (0.02, 3.87)	**BCG_INH**	0.69 (0.05, 7.84)	1.31 (0.20, 8.01)	0.79 (0.07, 8.40)	1.59 (0.17, 14.16)
0.86 (0.17, 4.63)	0.39 (0.03, 5.42)	1.45 (0.13, 18.85)	**BCG_MMC**	1.89 (0.25, 16.30)	1.15 (0.14, 10.37)	2.31 (0.45, 12.15)
0.45 (0.12, 1.67)	0.20 (0.02, 2.03)	0.76 (0.12, 4.91)	0.53 (0.06, 4.04)	**EPI**	0.61 (0.08, 4.36)	1.19 (0.20, 6.98)
0.74 (0.16, 3.45)	0.33 (0.03, 4.19)	1.26 (0.12, 14.80)	0.87 (0.10, 7.31)	1.64 (0.23, 12.63)	**GEM**	1.99 (0.32, 11.86)
0.37 (0.11, 1.25)	0.17 (0.02, 1.65)	0.63 (0.07, 5.75)	0.43 (0.08, 2.23)	0.84 (0.14, 5.06)	0.50 (0.08, 3.09)	**MMC**

Disease-specific Mortality					
**BCG**	0.94 (0.02, 62.49)	1.02 (0.34, 3.21)	0.66 (0.24, 1.50)	1.45 (0.61, 3.36)	1.04 (0.44, 2.39)
1.06 (0.02, 50.15)	**BCG_EPI**	1.08 (0.01, 58.75)	0.67 (0.01, 33.01)	1.51 (0.02, 77.52)	1.06 (0.01, 53.12)
0.98 (0.31, 2.95)	0.93 (0.02, 71.92)	**BCG_INH**	0.65 (0.13, 2.41)	1.41 (0.45, 4.02)	1.02 (0.22, 4.01)
1.52 (0.67, 4.24)	1.49 (0.03, 108.70)	1.54 (0.41, 7.68)	**BCG_MMC**	2.19 (0.68, 8.20)	1.56 (0.58, 5.14)
0.69 (0.30, 1.63)	0.66 (0.01, 46.96)	0.71 (0.25, 2.20)	0.46 (0.12, 1.48)	**EPI**	0.72 (0.22, 2.37)
0.96 (0.42, 2.30)	0.94 (0.02, 66.73)	0.98 (0.25, 4.45)	0.64 (0.19, 1.73)	1.40 (0.42, 4.63)	**MMC**

Fever					
**BCG**	0.40 (0.04, 2.88)	1.87 (0.23, 10.82)	0.61 (0.08, 2.16)	0.33 (0.03, 2.78)	**0.16 (0.04, 0.50)**
2.48 (0.35, 23.42)	**BCG_EPI**	4.39 (0.26, 71.94)	1.40 (0.08, 18.35)	0.78 (0.04, 17.48)	0.40 (0.03, 4.84)
0.54 (0.09, 4.42)	0.23 (0.01, 3.89)	**BCG_INH**	0.32 (0.04, 1.89)	0.18 (0.01, 3.79)	**0.09 (0.01, 0.97)**
1.64 (0.46, 12.65)	0.71 (0.05, 12.55)	3.10 (0.53, 26.47)	**EPI**	0.57 (0.04, 11.29)	0.26 (0.04, 2.67)
3.02 (0.36, 37.16)	1.29 (0.06, 28.39)	5.68 (0.26, 109.10)	1.76 (0.09, 26.83)	**GEM**	0.48 (0.04, 7.08)
**6.31 (2.01, 25.05)**	2.50 (0.21, 29.69)	**11.60 (1.03, 123.98)**	3.78 (0.37, 23.27)	2.06 (0.14, 27.16)	**MMC**

Cystitis					
**BCG**	0.68 (0.16, 2.59)	0.13 (0.01, 2.32)	0.34 (0.08, 1.31)	0.22 (0.02, 2.31)	0.79 (0.08, 6.14)
1.47 (0.39, 6.09)	**MMC**	0.19 (0.01, 2.49)	0.49 (0.07, 3.74)	0.32 (0.02, 5.47)	1.13 (0.08, 13.65)
7.72 (0.43, 159.74)	5.23 (0.40, 79.26)	**GEM**	2.59 (0.10, 71.24)	1.69 (0.04, 79.64)	5.95 (0.15, 231.17)
2.97 (0.76, 12.83)	2.03 (0.27, 15.33)	0.39 (0.01, 9.53)	**EPI**	0.65 (0.04, 11.41)	2.31 (0.27, 19.31)
4.56 (0.43, 47.81)	3.13 (0.18, 48.99)	0.59 (0.01, 23.00)	1.54 (0.09, 23.08)	BCG+EPI	3.49 (0.13, 80.12)
1.27 (0.16, 12.04)	0.88 (0.07, 12.54)	0.17 (0.00, 6.87)	0.43 (0.05, 3.76)	0.29 (0.01, 7.99)	**BCG + INH**

Haematuria					
**BCG**	0.31 (0.04, 2.28)	0.21 (0.01, 4.65)	0.19 (0.02, 1.17)	0.27 (0.01, 5.02)	0.55 (0.02, 16.14)
3.22 (0.44, 26.17)	**MMC**	0.68 (0.03, 15.37)	0.60 (0.03, 9.25)	0.85 (0.02, 32.72)	1.80 (0.03, 94.56)
4.75 (0.21, 104.85)	1.46 (0.07, 33.05)	**GEM**	0.87 (0.02, 27.84)	1.23 (0.02, 99.40)	2.61 (0.03, 233.17)
5.37 (0.86, 41.23)	1.66 (0.11, 29.78)	1.15 (0.04, 46.72)	**EPI**	1.43 (0.05, 53.28)	3.00 (0.10, 96.15)
3.76 (0.20, 80.76)	1.18 (0.03, 45.96)	0.81 (0.01, 64.93)	0.70 (0.02, 20.87)	**BCG + EPI**	2.09 (0.02, 194.73)
1.82 (0.06, 57.61)	0.56 (0.01, 28.65)	0.38 (0.00, 35.66)	0.33 (0.01, 9.75)	0.48 (0.01, 45.77)	**BCG + INH**

*BCG: Bacille Calmette Guerin; MMC: Mytomicin C; GEM: Gemcitabine; EPI: Epirubicin; INH: Isoniazid.

**Figure 6:**
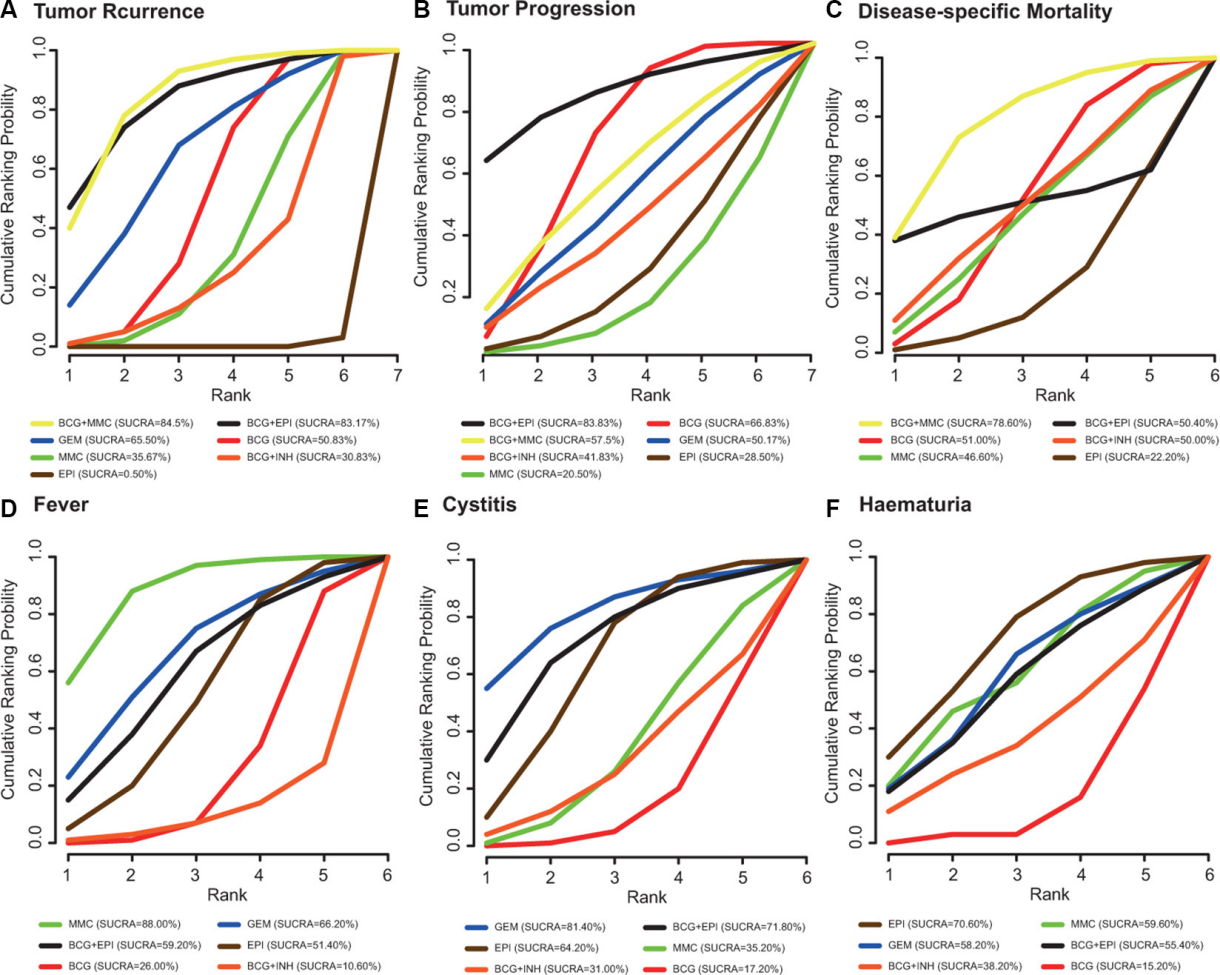
The corresponding results of SUCRA with respect to six endpoints (A) tumor recurrence; (B) tumor progression; (C) disease-specific mortality; (D) fever; (E) cystitis; (F) haematuria

The estimated probabilities and their corresponding SUCRA are displayed in Figure 6. Incorporating EPI or MMC into BCG (EPI+BCG, MMC+BCG) appeared to be the most preferred interventions with respect to TR, TP and DM. As for fever, cystitis and haematuria, BCG+EPI also exhibited a satisfied SUCRA value. Overall, incorporating EPI or MMC into BCG (EPI+BCG, MMC+BCG) was potentially more preferable than others when all of the six endpoints were taken into account.

## DISCUSSION

Instillation therapy with BCG has been considered as the standard treatment for patients with NMIBC [42]. The limitation of BCG motivates researchers to seek alternative chemotherapeutic agents that may have equivalent efficacy. We extracted data from 23 studies which incorporate a total of seven bladder intravesical instillation methods for NMIBC patients. Our objective is to determine whether these approaches have comparable efficacy with an increased safety and tolerability level. Direct evidence from conventional meta-analysis indicates that BCG is more preferred than EPI with respect to TR and TP. On the other hand, our network-meta analysis indicates that EPI is the worst with respect to TR. As shown by the corresponding ranking based on SUCRA values, both BCG + EPI and BCG + MMC have superior performance in comparison to other bladder intravesical instillations.

Adjuvant instillation of BCG has been verified as the most effective intravesical therapy that is able to prevent TR and progression [43–45]. Studies have concluded that the TR rate and TP rate can range from 23% to 49% and 4.8% to 25.6% over a five-year period among patients with intermediate and high-risk NMIBC [46, 47]. Furthermore, several studies reported that maintenance instillation of BCG is more effective than both chemotherapy and BCG monotherapy with respect to recurrence prevention for NMIBC patients [44, 48, 49]. However, maintenance instillation of BCG is not recommended for all NMIBC patients due to potential side effects [47].

EPI (4′-epidoxorubicin) is an antineoplastic agent which is derived from doxorubicin and it exerts antitumor effects by affecting DNA synthesis and such a mechanism is stimulated during the S phase of the cell cycle [50]. EPI has very limited effectiveness because it is a single chemotherapy agent against tumors and its effectiveness can be enhanced if combined with other chemotherapy agents [50]. A common conclusion obtained from both network-meta analysis and the ranking based on SUCRA values is that introducing BCG into EPI appears to be more effective than EPI monotherapy and this strategy is associated with a decrease in the risk of TR.

MMC is another chemotherapy agent which also inhibits DNA synthesis and there is no consensus about the difference between BCG+MMC and BCG monotherapy with respect to preventing TR and TP [51, 52]. Results from direct evidence showed that BCG + MMC are more effective than BCG monotherapy, and such a conclusion is supported by indirect evidence. Taking TR, TP and DM into consideration, BCG + MMC have several strengths with respect to these endpoints. A recent study also found that BCG + MMC appeared to be more effective than BCG or MMC monotherapy (5-year TR rate: 20.6% vs. 33.9%) [45].

The SUCRA ranking plot suggests that GEM may exhibit a safety profile with respect to the risk of fever, cystitis and haematuria. However, this trend is not reflected by our pairwise or network meta-analysis. We suspect this inconsistency for several reasons. Firstly, there may exit significant study heterogeneity which potentially causes the inconsistency between direct and indirect evidence. Secondly, the corresponding SUCRA values were produced by using the Bayesian Framework in conjunction with the MCMC sampling technique. Compared to the frequentist approach, the Bayesian framework does not lead to an unbiased estimator and such a characteristic may be reflected in the SUCRA values. The current literature indicates that GEM pyrimidine antimetabolite with approved efficacy and mild toxicity [53]. Nevertheless, the safety of GEM should be warranted in the future if it is used as a monotherapy or polytherapy for bladder patients. On the other hand, the current literature suggests that INH could cause chronic liver dysfunction and its application is restricted in clinical practices [27, 54] and our results also showed a worse performance of BCG + INH in TR.

This study is the first Bayesian network meta-analysis which simultaneously compares seven bladder intravesical instillation methods for patients with NMIBC. However, there are still some limitations affecting the results of our analysis. On one hand, there are large deviations in the sample size among different studies and such unbalanced sample size may have significant influence on the overall conclusions. On the other hand, we only analyzed the short-term effects of intravesical instillation methods on NMIBC patients due to the lack of evidence and thereby prognoses like survival rate of these approaches are still unclear.

In conclusion, combined intravesical instillation methods such as BCG + EPI and BCG + MMC exhibited outstanding efficacy for NIMBC patients. Nevertheless, future studies should further investigate the long-term effects of these intravesical instillation methods on NMIBC patients and the selection of appropriate methods should be validated by using well designed trials.

## MATERIALS AND METHODS

### Search strategy

Randomized control trials (RCTs) with “Bacillus Calmette - Guerin” or “mytomicin C” or “gemcitabine” or “epirubicin” or “isoniazid” matched with “bladder Cancer” was searched in PubMed, Embase, China National Knowledge Internet (CNKI), which were update to April, 2016 without any restriction on language. A manual search was also performed on the reference list of each relevant study, previous meta-analyses and earlier reviews of intravesical instillation therapies in relation to bladder tumor so that inappropriate omission can be prevented. The literature searching and retrieving process were carried out by two reviewers independently. All the arguments were solved by a third reviewer.

### Inclusion and exclusion criteria

Studies are eligible if: (1) their sample size were greater than 30 and (2) belonged to Randomized control trials (RCTs); (3) patients were above 18 years old and pathologically diagnosed with bladder cancer; (4) the efficacy of two or more intravesical instillation therapies (BCG, MMC, GEM, EPI, INH) were compared; (5) sufficient outcome data were provided. Articles are excluded if: (1) patients had history of muscle-invasive disease, upper urinary tract tumor or chronic urinary tract infection; (2) patients had other disease with immunodeficiency. The literature search, screen and selection flow chart was shown in Supplementary Figure S1.

### Outcome measures and data extraction

The following data was extracted from eligible studies, including gender, age, sample size, duration of follow-up, treatment and dosage. The effectiveness and tolerability of intravesical instillation therapies were assessed by using multiple endpoints including TR, TP, DM, fever, cystitis and haematuria. Data extraction was performed by two reviewers independently. Risk of complications such as fever, cystitis and haematuria were considered as secondary outcomes which evaluated the tolerability of intravesical instillation therapies.

### Statistical analysis

Conventional pair-wise meta-analysis was initially carried out and it incorporates direct evidence for each pair of treatment comparison. The fixed-effect model or random-effects model was implemented in pair-wise meta-analysis and the odds ratios (ORs) with their corresponding 95% confidence intervals (CI) were pooled in order to produce a summary effect size for each endpoint. Heterogeneity among studies was assessed by using the statistic of *I*^2^ and Q. No significant heterogeneity was suggested if *P* > 0.05 or *I*^2^ < 50% and thereby a fixed-effect model was selected. Otherwise, a random-effects model was taken into account due to the presence of significant heterogeneity.

Next, we carried out a network meta-analysis which is based on the Bayesian framework using R 3.2.3 software. Both direct and indirect evidence were synthesized from selected studies in order to produce summary statistics for each endpoint. Aside from that, intravesical instillation interventions are ranked by using the surface under the cumulative ranking curve (SUCRA) which produces a comprehensive ranking with respect to each endpoint. The corresponding ranking probabilities were defined as cumulative probabilities that each treatment being ranked as the first, second and so on. The larger SUCRA values, the more preferred ranking for each intravesical instillation intervention.

## SUPPLEMENTARY MATERIALS



## References

[1] Jemal A, Bray F, Center MM, Ferlay J, Ward E, Forman D (2011). Global cancer statistics. Cancer J Clin.

[2] Hayne D, Stockler M, McCombie SP, Chalasani V, Long A, Martin A, Sengupta S, Davis ID (2015). BCG+MMC trial: adding mitomycin C to BCG as adjuvant intravesical therapy for high-risk, non-muscle-invasive bladder cancer: a randomised phase III trial (ANZUP 1301). BMC cancer.

[3] Saika T, Tsushima T, Nasu Y, Miyaji Y, Saegusa M, Takeda K, Kumon H (2010). Two instillations of epirubicin as prophylaxis for recurrence after transurethral resection of Ta and T1 transitional cell bladder cancer: a prospective, randomized controlled study. World J Urol.

[4] Babjuk M, Burger M, Zigeuner R, Shariat SF, van Rhijn BW, Comperat E, Sylvester RJ, Kaasinen E, Bohle A, Palou Redorta J, Roupret M (2013). European Association of U. EAU guidelines on non-muscle-invasive urothelial carcinoma of the bladder: update 2013. Eur Urol.

[5] Chen SY, Du LD, Zhang YH (2010). Pilot study of intravesical instillation of two new generation anthracycline antibiotics in prevention of superficial bladder cancer recurrence. Chin Med J (Engl).

[6] Addeo R, Caraglia M, Bellini S, Abbruzzese A, Vincenzi B, Montella L, Miragliuolo A, Guarrasi R, Lanna M, Cennamo G, Faiola V, Del Prete S (2010). Randomized phase III trial on gemcitabine versus mytomicin in recurrent superficial bladder cancer: evaluation of efficacy and tolerance. J Clin Oncol.

[7] Braasch MR, Bohle A, O'Donnell MA (2008). Risk-adapted use of intravesical immunotherapy. BJU Int.

[8] Bassi P (2002). BCG (Bacillus of Calmette Guerin) therapy of high-risk superficial bladder cancer. Surg Oncol.

[9] Witjes JA, Hendricksen K (2008). Intravesical pharmacotherapy for non-muscle-invasive bladder cancer: a critical analysis of currently available drugs, treatment schedules, and long-term results. Eur Urol.

[10] Sylvester RJ, Oosterlinck W, Witjes JA (2008). The schedule and duration of intravesical chemotherapy in patients with non-muscle-invasive bladder cancer: a systematic review of the published results of randomized clinical trials. Eur Urol.

[11] Shang PF, Kwong J, Wang ZP, Tian J, Jiang L, Yang K, Yue ZJ, Tian JQ (2011). Intravesical Bacillus Calmette-Guerin versus epirubicin for Ta and T1 bladder cancer. Cochrane Database Syst Rev.

[12] Malmstrom PU (2004). Advances in intravesical therapy of urinary bladder cancer. Expert Rev Anticancer Ther.

[13] Gontero P, Marini L, Frea B (2005). Intravesical gemcitabine for superficial bladder cancer: rationale for a new treatment option. BJU Int.

[14] Nagel S, Califano R, Thatcher N, Blackhall F (2007). Gemcitabine and carboplatin in combination for the treatment of advanced, metastatic, non-small cell lung cancer. Expert Opin Pharmacother.

[15] Doi K, Ohchi T, Ogata K, Matsuo A, Kudoh K, Ohtao R, Muranaka T (2007). [Assessment of efficacy of arterial infusion chemotherapy with gemcitabine for four cases of advanced pancreatic cancer]. Gan to kagaku ryoho.

[16] Gazzaniga P, Silvestri I, Gradilone A, Scarpa S, Morrone S, Gandini O, Gianni W, Frati L, Agliano AM (2007). Gemcitabine-induced apoptosis in 5637 cell line: an *in-vitro* model for high-risk superficial bladder cancer. Anti-cancer drugs.

[17] Huncharek M, Geschwind JF, Witherspoon B, McGarry R, Adcock D (2000). Intravesical chemotherapy prophylaxis in primary superficial bladder cancer: a meta-analysis of 3703 patients from 11 randomized trials. J Clin Oncol.

[18] Okamura K, Ono Y, Kinukawa T, Matsuura O, Yamada S, Ando T, Fukatsu T, Ohno Y, Ohshima S, Nagoya University Urological Oncology G (2002). Randomized study of single early instillation of (2”R)-4′-O-tetrahydropyranyl-doxorubicin for a single superficial bladder carcinoma. Cancer.

[19] Witjes JA (2009). Topic issue on new treatments in bladder cancer. World J Urol.

[20] Solsona E, Madero R, Chantada V, Fernandez JM, Zabala JA, Portillo JA, Alonso JM, Astobieta A, Unda M, Martinez-Pineiro L, Rabadan M, Ojea A, Rodriguez-Molina J (2015). Sequential combination of mitomycin C plus bacillus Calmette-Guerin (BCG) is more effective but more toxic than BCG alone in patients with non-muscle-invasive bladder cancer in intermediate- and high-risk patients: final outcome of CUETO 93009, a randomized prospective trial. Eur Urol.

[21] Gontero P, Oderda M, Mehnert A, Gurioli A, Marson F, Lucca I, Rink M, Schmid M, Kluth LA, Pappagallo G, Sogni F, Sanguedolce F, Schiavina R (2013). The impact of intravesical gemcitabine and 1/3 dose Bacillus Calmette-Guerin instillation therapy on the quality of life in patients with nonmuscle invasive bladder cancer: results of a prospective, randomized, phase II trial. J Urol.

[22] Järvinen R, Kaasinen E, Rintala E (2012). Long-term results of maintenance treatment of mitomycin C or alternating mitomycin C and bacillus Calmette-Guérin instillation therapy of patients with carcinoma *in situ* of the bladder: A subgroup analysis of the prospective FinnBladder 2 study with a 17-year follow-up. Scand J Urol Nephrol.

[23] Hinotsu S, Akaza H, Naito S, Ozono S, Sumiyoshi Y, Noguchi S, Yamaguchi A, Nagamori S, Terai A, Nasu Y, Kume H, Tomita Y, Tanaka Y (2011). Maintenance therapy with bacillus Calmette-Guérin Connaught strain clearly prolongs recurrence-free survival following transurethral resection of bladder tumour for non-muscle-invasive bladder cancer. BJU Int.

[24] Oosterlinck W, Kirkali Z, Sylvester R, Silva FCD, Busch C, Algaba F, Collette S, Bono A (2011). Sequential intravesical chemoimmunotherapy with mitomycin C and bacillus Calmette-Guérin and with Bacillus Calmette-Guérin alone in patients with carcinoma *in situ* of the urinary bladder: Results of an EORTC genito-urinary group randomized phase 2 trial (30993). Eur Urol.

[25] Di Lorenzo G, Perdona S, Damiano R, Faiella A, Cantiello F, Pignata S, Ascierto P, Simeone E, De Sio M, Autorino R (2010). Gemcitabine versus bacille Calmette-Guerin after initial bacille Calmette-Guerin failure in non-muscle-invasive bladder cancer: a multicenter prospective randomized trial. Cancer.

[26] Porena M, Del Zingaro M, Lazzeri M, Mearini L, Giannantoni A, Bini V, Costantini E (2010). Bacillus Calmette-Guerin versus gemcitabine for intravesical therapy in high-risk superficial bladder cancer: a randomised prospective study. Urol Int.

[27] Sylvester RJ, Brausi MA, Kirkels WJ, Hoeltl W, Calais Da Silva F, Powell PH, Prescott S, Kirkali Z, van de Beek C, Gorlia T, de Reijke TM, Group EG-UTC (2010). Long-term efficacy results of EORTC genito-urinary group randomized phase 3 study 30911 comparing intravesical instillations of epirubicin, bacillus Calmette-Guerin, and bacillus Calmette-Guerin plus isoniazid in patients with intermediate- and high-risk stage Ta T1 urothelial carcinoma of the bladder. Eur Urol.

[28] Cai T, Nesi G, Tinacci G, Zini E, Mondaini N, Boddi V, Mazzoli S, Bartoletti R (2008). Can Early Single Dose Instillation of Epirubicin Improve Bacillus Calmette-Guerin Efficacy in Patients With Nonmuscle Invasive High Risk Bladder Cancer? Results From a Prospective, Randomized, Double-Blind Controlled Study. J Urol.

[29] Friedrich MG, Pichlmeier U, Schwaibold H, Conrad S, Huland H (2007). Long-Term Intravesical Adjuvant Chemotherapy Further Reduces Recurrence Rate Compared with Short-Term Intravesical Chemotherapy and Short-Term Therapy with Bacillus Calmette-Guérin (BCG) in Patients with Non-Muscle-Invasive Bladder Carcinoma. Eur Urol.

[30] Ojea A, Nogueira JL, Solsona E, Flores N, Gómez JMF, Molina JR, Chantada V, Camacho JE, Piñeiro LM, Rodríguez RH, Isorna S, Blas M, Martínez-Piñeiro JA (2007). A Multicentre, Randomised Prospective Trial Comparing Three Intravesical Adjuvant Therapies for Intermediate-Risk Superficial Bladder Cancer: Low-Dose Bacillus Calmette-Guerin (27 mg) versus Very Low-Dose Bacillus Calmette-Guerin (13. 5 mg) versus Mitomycin C. Eur Urol.

[31] Cheng CW, Chan SF, Chan LW, Chan CK, Ng CF, Cheung HY, Chan SY, Wong WS, Lai FM, To KF, Li ML (2005). Twelve-year follow up of a randomized prospective trial comparing bacillus Calmette-Guerin and epirubicin as adjuvant therapy in superficial bladder cancer. Int J Urol.

[32] de Reijke TM, Kurth KH, Sylvester RJ, Hall RR, Brausi M, van de Beek K, Landsoght KE, Carpentier P, European Organization for the R, Treatment of Cancer-Genito-Urinary G (2005). Bacillus Calmette-Guerin versus epirubicin for primary, secondary or concurrent carcinoma *in situ* of the bladder: results of a European Organization for the Research and Treatment of Cancer--Genito-Urinary Group Phase III Trial (30906). J Urol.

[33] Kaasinen E, Wijkström H, Malmström PU, Hellsten S, Duchek M, Mestad O, Rintala E (2003). Alternating mitomycin C, BCG instillations versus BCG alone in treatment of carcinoma *in situ* of the urinary bladder: A Nordic study. Eur Urol.

[34] Di Stasi SM, Giannantoni A, Stephen RL, Capelli G, Navarra P, Massoud R, Vespasiani G (2003). Intravesical electromotive mitomycin C versus passive transport mitomycin C for high risk superficial bladder cancer: a prospective randomized study. J Urol.

[35] van der Meijden AP, Brausi M, Zambon V, Kirkels W, de Balincourt C, Sylvester R, Members of the EG-UG (2001). Intravesical instillation of epirubicin, bacillus Calmette-Guerin and bacillus Calmette-Guerin plus isoniazid for intermediate and high risk Ta, T1 papillary carcinoma of the bladder: a European Organization for Research and Treatment of Cancer genito-urinary group randomized phase III trial. J Urol.

[36] Bilen CY, Özen H, Aki FT, Aygün C, Ekici S, Kendi S (2000). Clinical experience with BCG alone versus BCG plus epirubicin. Int J Urol.

[37] Ali-El-Dein B, Nabeeh A, Ismail EH, Ghoneim MA (1999). Sequential bacillus Calmette-Guerin and epirubicin versus bacillus Calmette-Guerin alone for superficial bladder tumors: A randomized prospective study. J Urol.

[38] Witjes JA, Meijden APMVD, Collette L, Sylvester R, Debruyne FMJ, Van Aubel A, Witjes WPJ, Boeken K, Van A, Carpentier De W, Oosterlinck Bouffioux (1998). Long-term follow-up of an eortc randomized prospective trial comparing intravesical bacille Calmette-Guerin-RIVM and mitomycin C in superficial bladder cancer. Urology.

[39] Melekos MD, Zarakovitis I, Dandinis K, Fokaefs E, Chionis H, Dauaher H, Barbalias G (1996). BCG versus epirubicin in the prophylaxis of multiple superficial bladder tumours: results of a prospective randomized study using modified treatment schemes. Int Urol Nephrol.

[40] Rintala E, Jauhiainen K, Kaasinen E, Nurmi M, Alfthan O, Hansson E, Juusela H, Kanerva K, Korhonen H, Permi J, Petäys P, Puolakka VM, Rajala P (1996). Alternating mitomycin C and bacillus Calmette-Guerin instillation prophylaxis for recurrent papillary (stages Ta to T1) superficial bladder cancer. J Urol.

[41] Lamm DL, Blumenstein BA, David Crawford E, Crissman JD, Lowe BA, Smith Jr JA, Sarosdy MF, Schellhammer PF, Sagalowsky AI, Messing EM, Loehrer P, Barton Grossman H (1995). Randomized intergroup comparison of bacillus calmette-guerin immunotherapy and mitomycin C chemotherapy prophylaxis in superficial transitional cell carcinoma of the bladder a southwest oncology group study. Urol Oncol.

[42] Hemdan T, Johansson R, Jahnson S, Hellstrom P, Tasdemir I, Malmstrom PU, Members of the Urothelial Cancer Group of the Nordic Association of U (2014). 5-Year outcome of a randomized prospective study comparing bacillus Calmette-Guerin with epirubicin and interferon-alpha2b in patients with T1 bladder cancer. J Urol.

[43] Sylvester RJ, van der MA, Lamm DL (2002). Intravesical bacillus Calmette-Guerin reduces the risk of progression in patients with superficial bladder cancer: a meta-analysis of the published results of randomized clinical trials. J Urol.

[44] Malmstrom PU, Sylvester RJ, Crawford DE, Friedrich M, Krege S, Rintala E, Solsona E, Di Stasi SM, Witjes JA (2009). An individual patient data meta-analysis of the long-term outcome of randomised studies comparing intravesical mitomycin C versus bacillus Calmette-Guerin for non-muscle-invasive bladder cancer. Eur Urol.

[45] Solsona E, Madero R, Chantada V, Fernandez JM, Zabala JA, Portillo JA, Alonso JM, Astobieta A, Unda M, Martinez-Pineiro L, Rabadan M, Ojea A, Rodriguez-Molina J (2015). Sequential combination of mitomycin C plus bacillus Calmette-Guerin (BCG) is more effective but more toxic than BCG alone in patients with non-muscle-invasive bladder cancer in intermediate- and high-risk patients: final outcome of CUETO 93009, a randomized prospective trial. Eur Urol.

[46] Fernandez-Gomez J, Madero R, Solsona E, Unda M, Martinez-Pineiro L, Gonzalez M, Portillo J, Ojea A, Pertusa C, Rodriguez-Molina J, Camacho JE, Rabadan M, Astobieta A (2009). Predicting nonmuscle invasive bladder cancer recurrence and progression in patients treated with bacillus Calmette-Guerin: the CUETO scoring model. J Urol.

[47] Brausi M, Oddens J, Sylvester R, Bono A, van de Beek C, van Andel G, Gontero P, Turkeri L, Marreaud S, Collette S, Oosterlinck W (2014). Side effects of Bacillus Calmette-Guerin (BCG) in the treatment of intermediate- and high-risk Ta, T1 papillary carcinoma of the bladder: results of the EORTC genito-urinary cancers group randomised phase 3 study comparing one-third dose with full dose and 1 year with 3 years of maintenance BCG. Eur Urol.

[48] Higashihara E, Nutahara K, Kojima M, Okegawa T, Miura I, Miyata A, Kato M, Sugisaki H, Tomaru T (1996). Significance of serum free prostate specific antigen in the screening of prostate cancer. J Urol.

[49] Bohle A, Bock PR (2004). Intravesical bacille Calmette-Guerin versus mitomycin C in superficial bladder cancer: formal meta-analysis of comparative studies on tumor progression. Urology.

[50] Cersosimo RJ, Hong WK (1986). Epirubicin: a review of the pharmacology, clinical activity, and adverse effects of an adriamycin analogue. J Clin Oncol.

[51] Kaasinen E, Wijkstrom H, Malmstrom PU, Hellsten S, Duchek M, Mestad O, Rintala E, Nordic Urothelial Cancer G (2003). Alternating mitomycin C, BCG instillations versus BCG alone in treatment of carcinoma *in situ* of the urinary bladder: a nordic study. Eur Urol.

[52] Oosterlinck W, Kirkali Z, Sylvester R, da Silva FC, Busch C, Algaba F, Collette S, Bono A (2011). Sequential intravesical chemoimmunotherapy with mitomycin C and bacillus Calmette-Guerin and with bacillus Calmette-Guerin alone in patients with carcinoma *in situ* of the urinary bladder: results of an EORTC genito-urinary group randomized phase 2 trial (30993). Eur Urol.

[53] Schlack K, Boegemann M, Steinestel J, Schrader AJ, Krabbe LM (2016). The safety and efficacy of gemcitabine for the treatment of bladder cancer. Expert Rev Anticancer Ther.

[54] Ojea A, Nogueira JL, Solsona E, Flores N, Gomez JM, Molina JR, Chantada V, Camacho JE, Pineiro LM, Rodriguez RH, Isorna S, Blas M, Martinez-Pineiro JA (2007). A multicentre, randomised prospective trial comparing three intravesical adjuvant therapies for intermediate-risk superficial bladder cancer: low-dose bacillus Calmette-Guerin (27 mg) versus very low-dose bacillus Calmette-Guerin (13. 5 mg) versus mitomycin C. Eur Urol.

