# Supplementary material for: Evaluating the efficacy and safety of intravesical chemotherapies for non-muscle invasive bladder cancer: a network meta-analysis
Source: Oncotarget. 2016 Oct 24;7(50):82567–79. doi: 10.18632/oncotarget.12856 (PMC5347714; doi:10.18632/oncotarget.12856)
Supplement: Supplementary file 1 [file oncotarget-07-82567-s001.pdf]

Evaluating the efficacy and safety of intravesical chemotherapies for non-muscle invasive bladder cancer: a network meta-analysis

Supplementary Materials

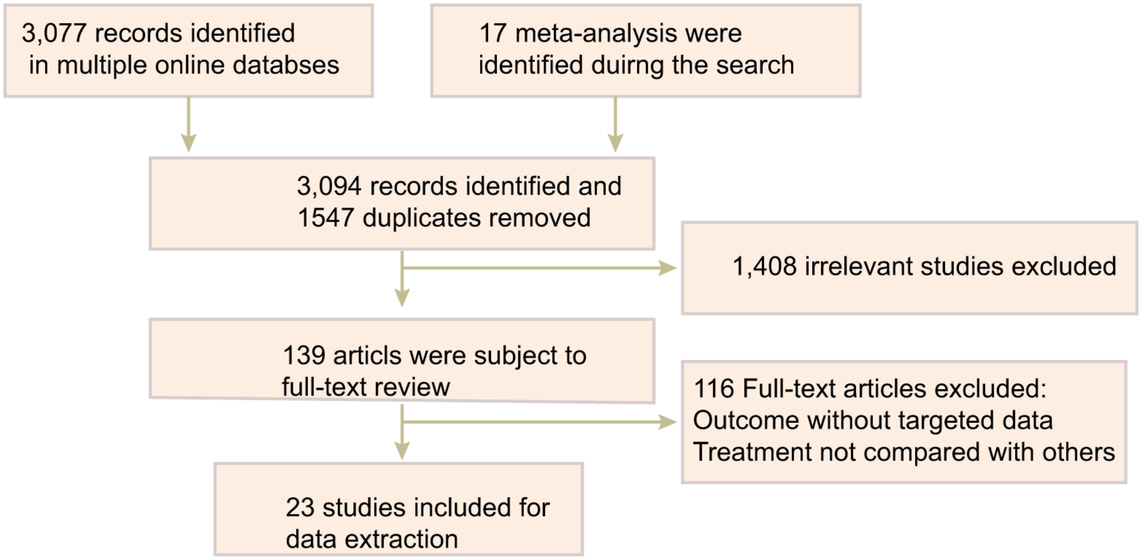

Supplementary Figure S1: The literature search, screen and selection flow chart.

Supplementary Table S1: Assessing the quality of included studies using the jadad scale

Author	Year	Randomization	Blinding	Description of Withdrawals
Solsona	2015	2	0	1
Gontero	2013	2	0	1
Järvinen	2012	2	0	0
Oosterlinck	2011	2	0	0
Hinotsu	2011	1	0	0
Sylvester	2010	1	0	0
Porena	2010	2	1	1
Di Lorenzo	2010	2	0	0
Addeo	2010	1	0	0
Cai	2008	1	2	0
Ojea	2007	1	0	1
Friedrich	2007	2	0	0
de Reijke	2005	1	0	0
Cheng	2005	1	0	0
Kaasinen	2003	1	0	1
Di Stasi	2003	2	0	0
Van Der Meijden	2001	1	2	0
Bilen	2000	1	0	0
Ali-El-Dein	1999	1	0	0
Witjes	1998	1	0	1
Rintala	1996	1	0	0
Melekos	1996	2	0	0
Lamm	1995	2	0	0

Randomization: randomization has been mentioned in the study and the randomization technique is appropriate (2); randomization has been mentioned in the study without details (1); no randomization mentioned (0).
Blinding: Blinding has been mentioned in the study and the blinding procedure was appropriate (2);
Blinding has been mentioned by the study without further details (1); no blinding procedures mentioned (0).
Withdrawals: The number of patients withdrawn from the study was mentioned (1); no withdrawals mentioned in the study (0).
Note: the maximum score of the Jaded Scale is 5.
